# ARHGEF4-mediates the actin cytoskeleton reorganization of hepatic stellate cells in 3-dimensional collagen matrices

**DOI:** 10.1080/19336918.2019.1594497

**Published:** 2019-03-24

**Authors:** Xiaowei Zhang, Lan Sun, Wei Chen, Shanna Wu, Yanmeng Li, Xiaojin Li, Bei Zhang, Jingyi Yao, Huan Wang, Anjian Xu

**Affiliations:** a Experimental Center, Beijing Friendship Hospital, Capital Medical University, Beijing, China; b State Key Laboratory of Bioactive Substance and Function of Natural Medicines, Institute of Materia Medica, Chinese Academy of Medical Sciences & Peking Union Medical College, Beijing, China; c Department of Pathology, Beijing Friendship Hospital, Capital Medical University, Beijing, China; d Clinical Laboratory Center, Beijing Friendship Hospital, Capital Medical University, Beijing, China

**Keywords:** 3D collagen matrices, hepatic stellate cell, actin cytoskeleton reorganization, ARHGEF4, migration

## Abstract

The actin cytoskeleton of hepatic stellate cells (HSCs) is reorganized when they are cultured in 3D collagen matrices. Here, we investigated the molecular mechanism of actin cytoskeleton reorganization in HSCs cultured in 3D floating collagen matrices (FCM) compared to those on 2D polystyrene surfaces (PS). First, we found that the generation of dendritic cellular processes was controlled by Rac1. Next, we examined the differential gene expression of HSCs cultured on 2D PS and in 3D FCM by RNA-Seq and focused on the changes of actin cytoskeleton reorganization-related molecular components and guanine nucleotide exchange factors (GEFs). The results showed that the expression of genes associated with actin cytoskeleton reorganization-related cellular components, filopodia and lamellipodia, were significantly decreased, but podosome-related genes was significantly increased in 3D FCM. Furthermore, we found that a Rac1-specific GEF, ARHGEF4, played roles in morphological changes, migration and podosome-related gene expression in HSCs cultured in 3D FCM.

**Abbreviations**: 2D PS: 2-dimensional polystyrene surface; 3D FCM: 3-dimensional floating collagen matrices; ARHGEF4: Rho guanine nucleotide exchange factor 4; ARHGEF6: Rho guanine nucleotide exchange factor 6; GEF: guanine nucleotide exchange factor; HSC: hepatic stellate cell

## Introduction

Hepatic stellate cells (HSCs), located in the sinusoidal space of Disse between endothelial cells and parenchymal cells, have been found to form many long cellular processes that surround the hepatic sinusoids *in vivo* [1]. However, HSCs show a flattened, fibroblast-like shape with well-developed lamellipodia and actin stress fibers when cultured on a 2D polystyrene surface (PS) or on a type I collagen-coated surface, suggesting the regulation of HSC morphology and function by extracellular matrix (ECM) components in the perisinusoidal space of Disse [2–4]. In fact, HSCs exhibit an *in vivo* morphology with long cellular processes when cultured in 3D collagen matrices and do not form clear lamellipodia and actin stress fibers [3,5–7]. Thus, HSCs cultured in 3D collagen matrices may be better able to mimic the morphology and function of HSCs in an *in vivo* environment.

The morphological changes of cells are usually caused by actin cytoskeleton reorganization, especially the formation of lamellipodia and actin stress fibers, which are closely related to actin cytoskeleton reorganization [8–11]. Mechanistically, small G proteins (also known as GTPases), such as Rac1 and RhoA, are particularly important in actin cytoskeleton reorganization [12,13]. These GTPases are regulated by guanine nucleotide exchange factors (GEFs), which activate GTPases by facilitating the exchange of GDP for GTP [14]. Although the morphological change of HSCs cultured in 3D collagen matrices has been known for over a decade, the underlying mechanism of the HSC morphological change and the molecular mechanism of actin cytoskeleton reorganization in 3D culture remain to be elucidated.

In the current research, we studied the actin cytoskeleton-related molecular change of HSCs cultured in 3D floating collagen matrices (FCM) compared to 2D PS by using RNA-Seq and focused on the changes to GEFs as well as their roles in the morphological changes of HSCs cultured in 3D FCM. The results showed that Rho guanine nucleotide exchange factor 4 (ARHGEF4) might play an important role in the morphological changes and actin cytoskeleton reorganization of HSCs cultured in 3D FCM.

## Results

### Hepatic stellate cells cultured in 3D floating collagen matrices exhibited different cell morphologies

**Figure 1.**
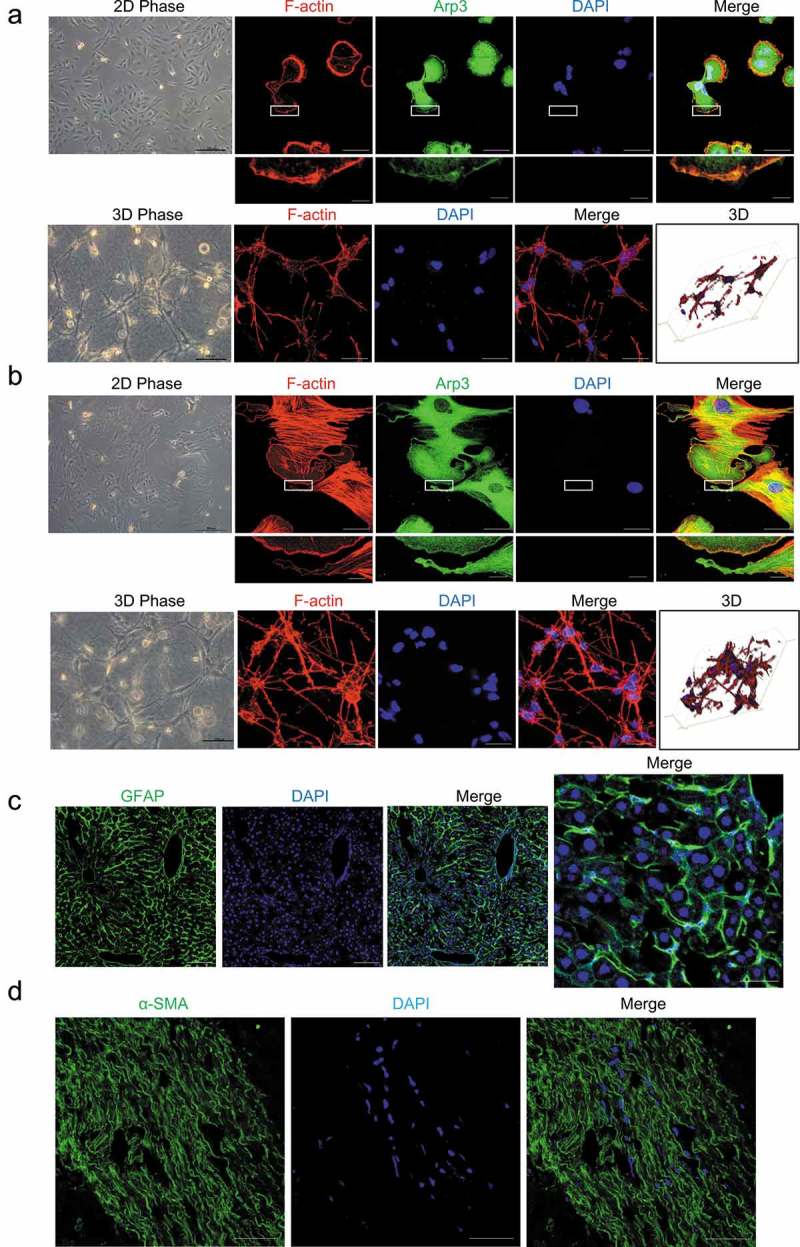
Cell morphology of hepatic stellate cells (HSCs). (a and b). Human LX-2 HSCs (A) and rat HSCs (B) cultured on a 2D polystyrene surface (2D PS) showed a flattened morphology without cellular processes and were spread out with well-developed lamellipodia and actin stress fibers (top). LX-2 cells and rat HSCs were stained with rhodamine-conjugated phalloidin (F-actin), an antibody against Arp3 (a marker of lamellipodia) and DAPI (nuclear DNA). Human LX-2 HSCs (A) and rat HSCs (B) cultured in 3D floating collagen matrices (3D FCM) exhibited stellate or dendritic morphologies and long, slender cellular processes (bottom). LX-2 cells and rat HSCs cultured in 3D FCM were stained with rhodamine-conjugated phalloidin (F-actin). Images were photographed ≥ 150 μm away from the surface with a confocal microscope. (c). Immunofluorescence staining for GFAP (green) in the liver tissues from mice injected with CCl_4_ revealed that the GFAP-positive cells, HSCs, had expanded extended cellular processes along the sinusoids, which is similar to the phenotype in 3D FCM. (d). Immunofluorescence staining for α-SMA (green) of activated HSCs in human fibrosis liver showed long cellular processes along the fibrotic septa. Scale bars = 40 and 5 μm (insets).

Human HSCs, as well as primary rat HSCs, cultured on a 2D polystyrene surface (PS), showed a flattened shape without cellular processes. Immunofluorescence staining revealed that the cells cultured on 2D PS were spread out with well-developed lamellipodia and actin stress fibers (Figure 1(a)). However, hepatic stellate cells cultured in 3D floating collagen matrices (FCM) exhibited stellate or dendritic shapes and long, slender cellular processes (Figure 1(b)). This morphology is closer to the *in situ* morphology, as HSCs are located in the space of Disse and possess cytoplasmic processes that extend along the sinusoids *in vivo*. In fact, *in situ* immunofluorescence staining of GFAP in mouse liver revealed that the GFAP-positive cells, HSCs, had extended cellular processes along the sinusoids, which is similar to the phenotype of HSCs cultured in 3D FCM (Figure 1(c)). In addition, α-SMA staining of activated HSCs in human liver fibrosis also showed long cellular processes along the fibrotic septa (Figure 1(d)). Thus, HSCs cultured in 3D FCM may be better able to mimic the *in vivo* morphology of HSCs.

### The generation of dendritic cellular processes was controlled by Rac1

**Figure 2.**
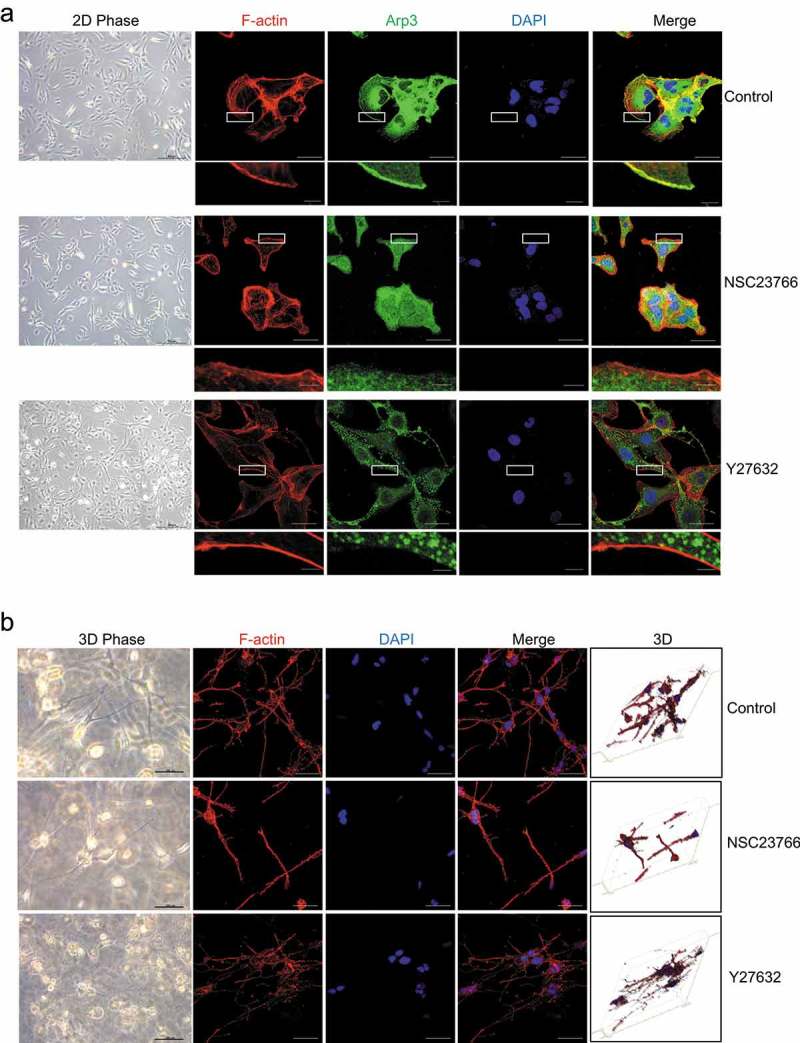
The effect of a Rho inhibitor on the cell morphology of HSCs. (a). The effect of the Rac1 inhibitor NSC23766 and the RhoA inhibitor Y27632 on the cell morphology of HSCs cultured on 2D PS. LX-2 cells were left untreated (top) or treated with NSC23766 (20 μM) for 1 h (middle) or Y27632 (10 nM) for 1 h (bottom) and then immunostained with rhodamine-conjugated phalloidin (F-actin), an antibody against Arp3 (a marker of lamellipodia) and DAPI (nuclear DNA). (b). The effect of the Rac1 inhibitor NSC23766 and the RhoA inhibitor Y27632 on the cell morphology of HSCs cultured in 3D FCM. LX-2 and rat HSCs cultured in 3D FCM were left untreated (up) or treated with NSC23766 (20 μM) for 1 h (middle) or Y27632 (10 nM) for 1 h (bottom) and then immunostained with rhodamine-conjugated phalloidin (F-actin). Images were photographed ≥ 150 μm away from the surface with a confocal microscope. Scale bars = 40 and 5 μm (insets).

To explore the mechanism of cellular process generation, we investigated the effect of the small GTPases Rac1 and RhoA, which are important small GTPases regulating lamellipodia and actin stress fibers on 2D surfaces, respectively, on cellular process generation. As shown in Figure 2(a), the Rac1 inhibitor NSC23766 suppressed lamellipodium formation in HSCs cultured on 2D PS, while the RhoA inhibitor Y27632 inhibited actin stress fiber organization. However, in 3D FCM, Rac1 inhibitor NSC23766-treated HSCs exhibited a dramatic decrease in cellular processes, whereas Y27632 did not affect cellular processes; however, the dendritic cellular processes were thinner than the controls (Figure 2(b)). Thus, Rac1 and RhoA might use different mechanisms to regulate the actin cytoskeleton structure of HSCs cultured in 3D FCM. In particular, Rac1 controlled the number of cellular processes of HSCs cultured in 3D FCM, while RhoA controlled the thickness of cellular processes.

### RNA-Seq of HSCs cultured on 2D PS and in 3D FCM

**Figure 3.**
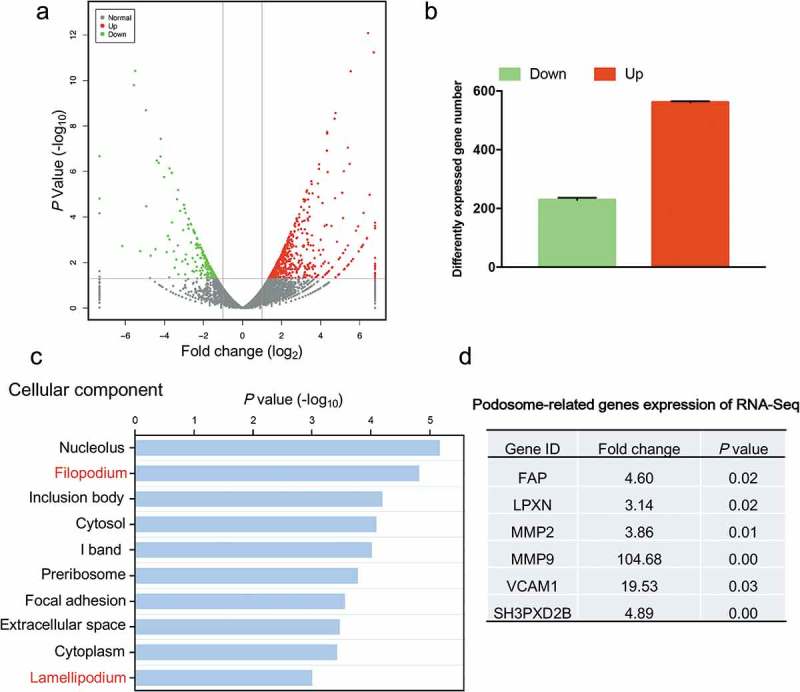
Gene expression analysis of HSCs cultured on 2D PS and in 3D FCM by RNA-Seq. (a). Volcano plot of the gene expression values. Red, gene expression upregulated in HSCs cultured in 3D FCM. Green, gene expression downregulated in HSCs cultured in 3D FCM. (b). The number of differentially expressed genes in Figure 3(a). (c). The top 10 significant signals from the cellular component of Go-enrichment analysis of downregulated genes. (d). The gene expression of podosome-related genes from the RNA-Seq data.

To further elucidate the molecular changes underlying the cell morphological differences between HSCs cultured on 2D PS and in 3D FCM, we analyzed the gene expression profiles of human LX-2 HSCs cultured on 2D PS and in 3D FCM by RNA-Seq (Figure 3(a)). Differential expression analysis resulted in 787 coding genes with an FDR-corrected *p*-value < 0.05 and fold change > 2 (Figure 3(b)). To identify the primary molecular events in actin cytoskeletal reorganization, we performed Go-enrichment analysis to reveal changes in actin cytoskeleton components. Notably, in the top ten cellular component categories of downregulated genes (2D PS *vs* 3D FCM), actin cytoskeletal reorganization-related cellular components, filopodium and lamellipodium were listed (Figure 3(c)). However, we noted that a number of genes related to podosomes were significantly increased in 3D FCM; podosomes are actin-based cellular structures (similar to filopodia and lamellipodia in 2D) and degrade ECM to facilitate cell migration in 3D or tissue microenvironments (Figure 3(d)). Thus, the actin cytoskeleton of HSCs cultured in 3D FCM underwent significant reorganization compared to 2D PS and presented elevated expression of podosome-related genes.

### ARHGEF4 and ARHGEF6 were significantly upregulated in HSCs cultured in 3D FCM

**Figure 4.**
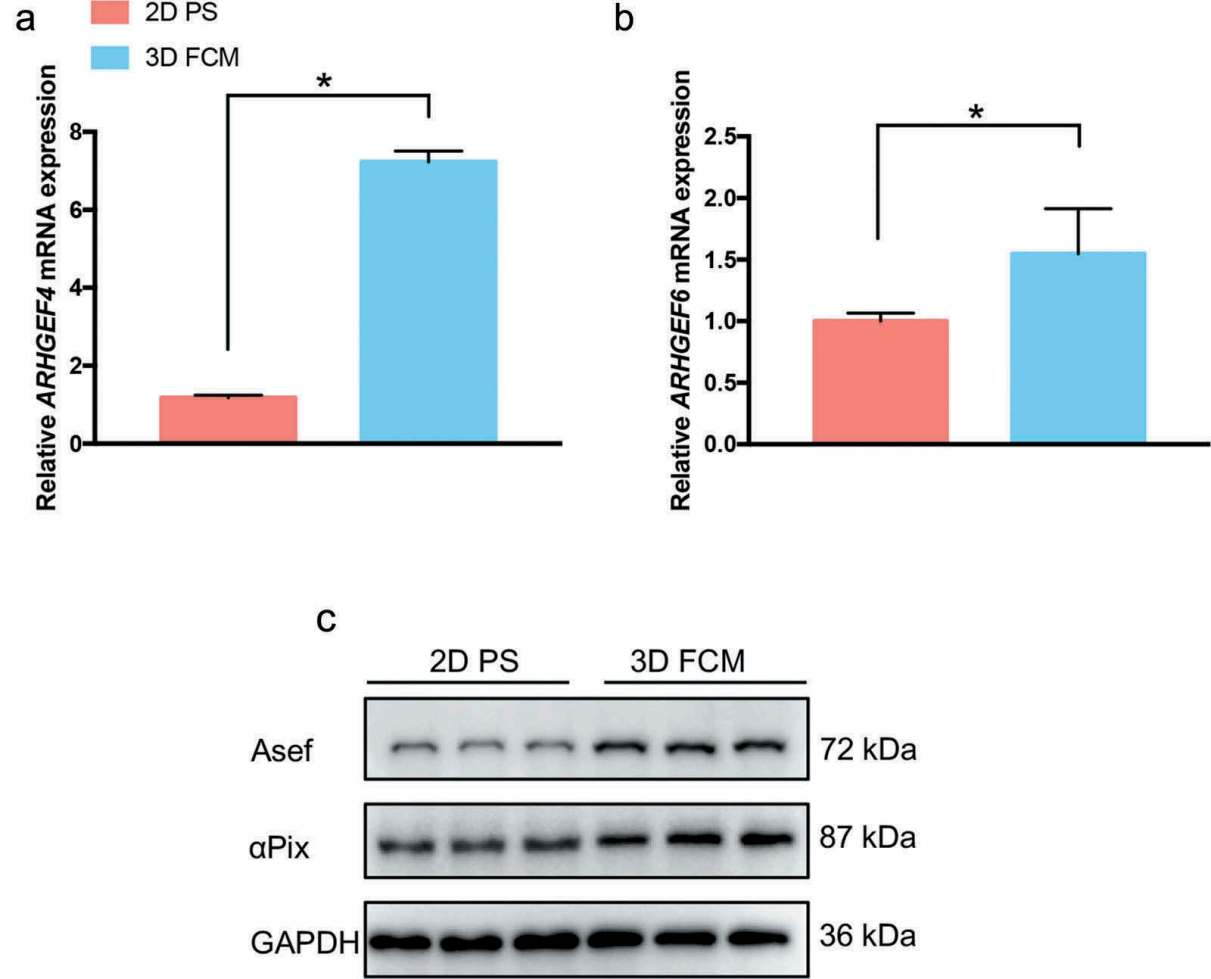
The expression of ARHGEF4 and ARHGEF6 was significantly upregulated in HSCs cultured in 3D FCM. (a). The significantly upregulated expression of ARHGEF4 mRNA in HSCs cultured in 3D FCM was confirmed by real-time PCR. LX-2 cells were cultured on 2D PS or in 3D FCM, then total RNA was isolated, and real-time PCR was performed to detect ARHGEF4 mRNA expression. *p < 0.05, data are the means ± SD. (b). The upregulated expression of ARHGEF6 mRNA in HSCs cultured in 3D FCM was confirmed by real-time PCR. LX-2 cells were cultured on 2D PS or in 3D FCM, then total RNA was isolated, and real-time PCR was performed to detect ARHGEF6 mRNA expression. *p < 0.05, data are the means ± SD. (c). The upregulated expression of Asef (ARHGEF4) protein and αPix (ARHGEF6) protein were confirmed by Western blotting. LX-2 cells were cultured on 2D PS or in 3D FCM for 1 d, and the proteins were obtained by treatment with lysis buffer or collagenase before lysis buffer for cells cultured in 3D FCM. Western blotting was performed to detect the protein expression of Asef and αPix.

The activity of Rac1 controls actin cytoskeletal reorganization, and Rac1 indeed regulated the generation of dendritic cellular processes in HSCs cultured in 3D FCM in our study (Figure 2(b)). Most importantly, many Rac1-regulated genes (specifically, filopodium-, lamellipodium- and podosome-related genes) showed significant changes between HSCs cultured on 2D PS and in 3D FCM (Figure 3(c,d)). Therefore, to reveal the mechanism of Rac1-mediated generation of dendritic cellular processes, we focused on the expression changes of guanine nucleotide exchange factors (GEFs), which regulate Rac1 GTPase activity. Among the GEFs, we found two genes with significantly altered expression: ARHGEF4 and ARHGEF6, which encode Rho guanine nucleotide exchange factor 4 (Asef) and Rho guanine nucleotide exchange factor 6 (COOL-2/αPix), respectively (Table 1). To confirm the results of RNA-Seq, we performed RT-PCR and Western blotting to detect the expression of ARHGEF4 and ARHGEF6 in LX-2 cells cultured on 2D PS and in 3D FCM. As a result, both ARHGEF4 and ARHGEF6 showed increased expression in 3D FCM (Figure 4(a-c)).

**Table 1.** Gene expression values from RNA-Seq.

Gene_ID	Fold change	*P* value
ARHGEF1	0.94	0.8895
ARHGEF2	1.26	0.6395
ARHGEF3	2.60	0.1211
ARHGEF4	10.79	0.0112
ARHGEF5	0.98	0.9859
ARHGEF6	11.21	0.0018
ARHGEF7	1.08	0.8741
ARHGEF9	1.04	0.9397
ARHGEF10	0.87	0.7668
ARHGEF10L	1.33	0.5761
ARHGEF11	0.86	0.7542
ARHGEF12	1.12	0.8303
ARHGEF16	1.31	0.7547
ARHGEF17	0.63	0.3584
ARHGEF18	0.69	0.4222
ARHGEF19	1.16	0.8190
ARHGEF25	1.93	0.1622
ARHGEF26	0.67	0.4667
ARHGEF28	1.04	0.9436
ARHGEF33	12.09	0.2051
ARHGEF35	0.33	0.3795
ARHGEF37	3.13	0.2191
ARHGEF39	0.68	0.5558
ARHGEF40	1.16	0.7567

### Knockdown of ARHGEF4 decreased the dendritic cellular processes of HSCs cultured in 3D FCM

**Figure 5.**
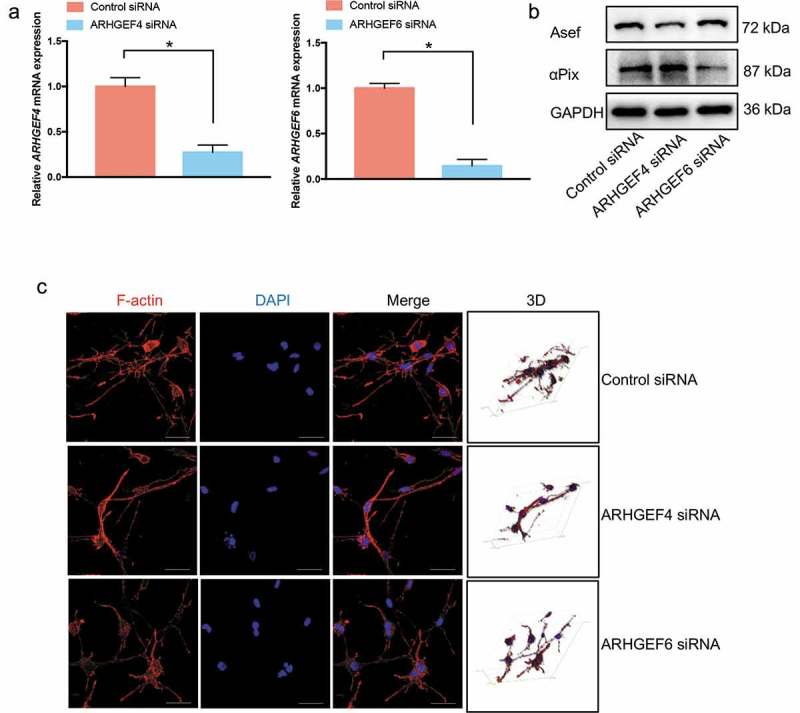
The effect of ARHGEF4 or ARHGEF6 knockdown on the dendritic cellular process formation of HSCs in 3D FCM. (a). The mRNA expression levels of ARHGEF4 (left) and ARHGEF6 (right) after RNA-mediated interference were detected by real-time PCR. *p < 0.05, data are the means ± SD. (b). The protein expression levels of Asef (ARHGEF4) and αPix (ARHGEF6) after RNA-mediated interference were detected by Western blotting. (c). The cell morphology of HSCs after ARHGEF4 or ARHGEF6 knockdown. LX-2 cells were treated with ARHGEF4 siRNA or ARHGEF6 siRNA for 2 d and cultured in 3D FCM for another day. Then, the cells were stained with rhodamine-conjugated phalloidin (F-actin). Images were photographed ≥ 150 μm away from the bottom with a confocal microscope. Scale bars = 40 μm.

To demonstrate the role of ARHGEF4 and ARHGEF6 in the dendritic cellular process generation of HSCs cultured in 3D FCM, we used RNA-mediated interference (RNAi) to knock down the expression of ARHGEF4 or ARHGEF6 (Figure 5(a,b)) and test the function of ARHGEF4 or ARHGEF6 in cell morphology and actin cytoskeletal reorganization. As expected, loss of ARHGEF4 resulted in HSCs with a decreased number of dendritic cellular processes in 3D FCM, similar to the results observed with the NSC23766 Rac1 inhibitor treatment. However, knockdown of ARHGEF6 did not significantly influence the number of HSC dendritic cellular processes when cultured in 3D FCM (Figure 5(c)). Thus, only ARHGEF4 exhibited a dendritic cellular process-regulating function in 3D FCM-cultured HSCs. Therefore, ARHGEF4 might play an important role in HSC 3D cell morphology.

### Knockdown of ARHGEF4 decreased the migration of HSCs in 3D FCM

**Figure 6.**
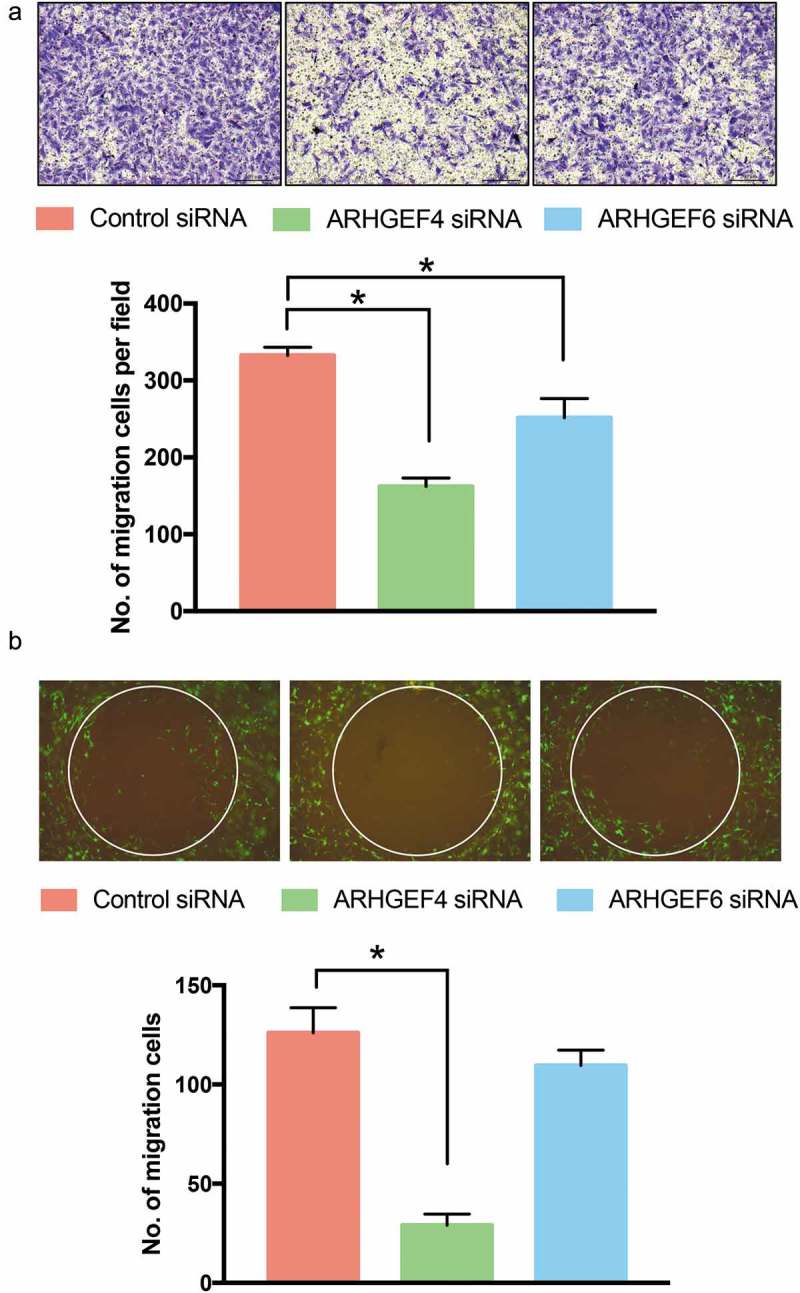
The effect of ARHGEF4 or ARHGEF6 knockdown on the cell migration of HSCs. (a). The cell migration of HSCs after ARHGEF4 or ARHGEF6 knockdown was evaluated by transwell assay. The number of migrated cells was counted in 4 random fields. (n = 4 from 3 independent experiments). *p < 0.05, data are the means ± SD. (b). The cell migration of HSCs after ARHGEF4 or ARHGEF6 knockdown was evaluated by 3D collagen invasion assay. Stably transfected pEGF LX-2 cells were treated with ARHGEF4 siRNA or ARHGEF6 siRNA for 2 d, and a 3D collagen invasion assay was performed by using an OrisTM 3D Embedded Invasion assay kit. The number of migrated cells was counted in 4 repeated experiments. *p < 0.05, data are the means ± SD.

Next, we wondered whether ARHGEF4 could regulate the migration of HSCs because the activity of Rac1 is closely related to cell migration. Therefore, we investigated the cell migration of HSCs in a transwell assay after ARHGEF4 and ARHGEF6 knockdown. The results showed that ARHGEF4 knockdown significantly decreased the motility of HSCs; however, ARHGEF6 knockdown only slightly decreased the motility of HSCs (Figure 6(a)). To reveal the effect of ARHGEF4 on cell motility in 3D cultures, we performed a 3D invasion assay. Notably, only ARHGEF4 knockdown significantly inhibited the 3D migration of HSCs, which indicated that ARHGEF4 could regulate the 3D cell motility of HSCs (Figure 6(b)).

### Knockdown of ARHGEF4 decreased the expression of podosome-related genes

**Figure 7.**
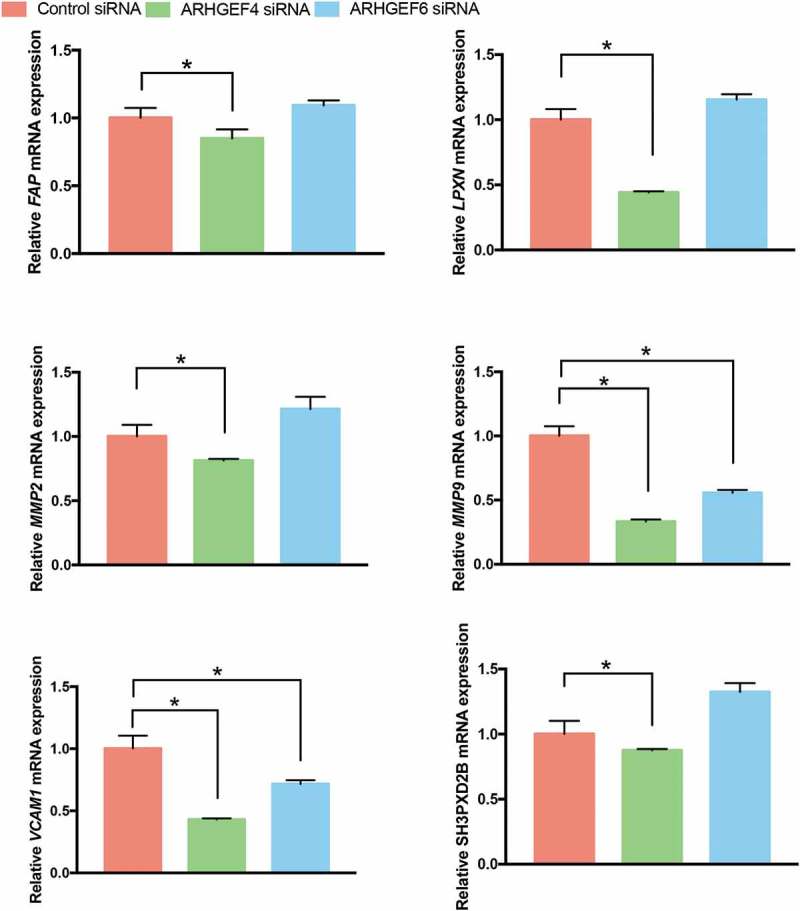
The mRNA expression of podosome-related genes after ARHGEF4 or ARHGEF6 knockdown in LX-2 cells cultured in 3D FCM. LX-2 cells were treated with ARHGEF4 siRNA or ARHGEF6 siRNA for 2 d and cultured in 3D FCM for another day. Total RNA was then isolated, and real-time PCR was performed to detect the mRNA expression of podosome-related genes. *p < 0.05, data are the means ± SD.

Based on our RNA-Seq results, the actin cytoskeletal reorganization in 3D FCM was characterized by the upregulation of podosome formation-related genes (Figure 3(d)). Thus, we speculated that the altered cell morphology and actin cytoskeletal reorganization caused by ARHGEF4 knockdown might also be due to the altered expression of podosome-related genes. Therefore, we investigated the expression of podosome-related genes after ARHGEF4 knockdown using real-time PCR. As a result, the expression of all podosome-related genes altered in 3D FCM significantly decreased after ARHGEF4 knockdown. However, only the expression of MMP9 and VCAM1 exhibited a significant reduction in expression after ARHGEF6 knockdown (Figure 7). These results confirmed the important role of ARHGEF4 in HSC 3D actin cytoskeleton reorganization and migration.

## Discussion

HSCs cultured on 2D polystyrene surfaces (2D PS), such as ordinary cell culture dishes, exhibit a fibroblast-like or flattened cell shape without cellular processes [2,3]. HSCs are known to possess many long cellular processes surrounding hepatic sinusoids *in vivo* [7]. In fact, it was reported decades ago that cultured HSCs respond to the ECM components used as substrates, altering their morphology, proliferation and function [4]. Thus, HSCs seem to lose their *in vivo* morphology under normal 2D culture conditions.

The 3D collagen culture technique is currently the best and most convenient way to mimic the *in vivo* environment of HSCs. Collagen is the main ECM component in normal liver, and in fibrotic liver, collagen type I secreted by activated HSCs is the main component of scars [15,16]. Many studies have used 3D collagen culture to investigate the morphology of HSCs and have found that cytoskeletal reorganization is involved in the alteration of HSC morphology [2–4,6]. In accordance with previous studies, we also found morphological changes in HSCs cultured in 3D FCM compared to 2D PS. In our study, we used a 3D FCM method wherein newly polymerized matrices are released and allowed to float in the medium to culture HSCs, which were introduced as the *in vitro* dermal component and combined with epidermal cells to create a living skin equivalent [17]. The 3D culture model in our study is most similar to the *in vivo* conditions; for the less similar, restrained method, where collagen matrices are attached to dishes, cells in FCM can project an elaborate dendritic network of extensions interconnected by gap junctions, and the matrix does not develop tension and the cells remain mechanically unloaded [18].

Although actin cytoskeleton reorganization results in an alteration of HSC morphology in 3D culture, the underlying mechanism of the HSC morphological change and the molecular mechanism remain unknown. The small G proteins Rac1 and RhoA are key factors that regulate actin cytoskeleton reorganization and cell migration [13,14]. In our study, we found that inhibition of Rac1, but not RhoA, activity decreased the cellular processes of HSCs cultured in 3D FCM, suggesting that the activity of Rac1 might mainly determine the number of cellular processes formed by HSCs cultured in 3D FCM. Therefore, we performed an RNA-Seq analysis to reveal the molecular changes of HSCs cultured in 3D FCM compared to 2D PS and focused on alterations of actin cytoskeleton reorganization-related cellular components and guanine nucleotide exchange factors (GEFs), which could activate Rho GTPase. The results showed that the expression of filopodium- and lamellipodium-related genes, which are actin-based cellular structures found in cells cultured in 2D, were significantly decreased in cells cultured in 3D FCM. However, the expression of podosome-related genes, which are actin-based cellular processes of the plasma membrane restricted to the ventral surface of cells cultured on 2D [19], was significantly upregulated.

Podosomes are actin-based dynamic cellular processes of the plasma membrane that represent the sites of attachment to and degradation of the extracellular matrix [19]. Podosomes have morphological characteristics distinct from other cellular processes, such as filopodia and lamellipodia [20]. In 2D culture, unlike filopodia and lamellipodia, which are located at the leading edge of the cells, the formation of podosomes is restricted to the ventral surface of the cells, which contacts the ECM [21,22]. In 3D culture, many studies consider the long cellular processes, which many mesenchymal cells and cancer cells form in 3D culture, to be podosomes because they contain podosome-associated proteins and have the ability to degrade ECM [23–25]. In our study, we speculate that the cellular processes of HSCs formed in 3D FCM culture are probably podosomes based on the structural characteristics and gene expression.

In addition, the expression of the guanine nucleotide exchange factors ARHGEF4 and ARHGEF6 was significantly increased in 3D FCM cultured HSCs compared to 2D PS cultured HSCs. Therefore, we speculate that the cellular processes of HSCs cultured in 3D FCM may be regulated by ARHGEF4 and ARHGEF6, and the podosome-related genes are downstream of ARHGEF4 (Asef) and ARHGEF6 (αPix). To test our hypothesis, the expression of ARHGEF4 or ARHGEF6 was inhibited using siRNA, and the effect of ARHGEF4 or ARHGEF6 knockdown on the formation of cellular processes and migration of HSCs cultured in 3D FCM were investigated. The results showed that only ARHGEF4 regulated cellular process formation and migration of HSCs cultured in 3D FCM, suggesting that ARHGEF4 is probably the main GEF involved in morphological changes of HSCs cultured in 3D FCM. In fact, the expression of all podosome-related genes upregulated in 3D FCM-cultured HSCs decreased after ARHGEF4 knockdown, confirming the important function of ARHGEF4 on actin cytoskeleton reorganization and migration of HSCs cultured in 3D.

ARHGEF4 (Asef) is a Rac1-specific GEF that contains a Dbl homology (DH) domain and exhibits GEF activity [26]. Previous studies have reported that Asef could mediate cell flattening, membrane ruffling and lamellipodium formation in cells cultured on 2D PS [27]. However, the function of Asef on actin cytoskeleton reorganization and migration of cells cultured in 3D has not been reported. In our study, we validated the function of Asef in 2D cell migration. Importantly, we extended the function of Asef to cells cultured in 3D, specifically, the important role in the morphological change and migration of HSCs cultured in 3D FCM.

Taken together, our results show that HSCs cultured in 3D FCM exhibit different gene expression profiles compared to those cultured on 2D PS. In particular, morphological changes in HSCs cultured in 3D FCM are probably due to alterations in the actin cytoskeleton structure and related gene expression levels. Most importantly, the regulation of actin cytoskeleton reorganization and migration in 3D culture shows different molecular mechanisms compared to 2D culture.

## Materials and methods

### Cultivation of human and rat HSCs

The human HSC cell line LX-2 [28] and rat HSCs, isolated as previously described [29], were cultured in minimum Eagle’s medium (MEM, Sigma, USA) containing 10% (v/v) fetal bovine serum, 100 U/mL penicillin, and 100 mg/mL streptomycin at 37 °C in a humidified atmosphere of 95% air and 5% CO_2_.

Human fibrotic liver samples were obtained from patients undergoing surgical hepatectomy and assessed by pathological examination of hematoxylin and eosin (H&E) and Masson’s Trichrome staining [29]. The study protocol was approved by the local ethics committee (Beijing Friendship Hospital, Capital Medical University, Beijing, China), and the samples were collected from subjects who provided informed consent for his or her tissue to be used for research purposes.

### Antibodies and reagents

The anti-αPix antibody was purchased from Cell Signaling (MA, USA). The anti-Asef antibody was purchased from Proteintech (Rosemont, USA). The anti-α-SMA antibody was purchased from Abcam (Cambridge, UK). The anti-GFAP antibody was purchased from Merck Serono (Darmstadt, Germany). The anti-Arp3 and anti-GAPDH antibodies were purchased from Santa Cruz Biotechnology (CA, USA). Anti-mouse Alex 488 and anti-rabbit Alex 488-conjugated secondary antibodies were purchased from Invitrogen (CA, USA). Horseradish peroxidase (HRP)-conjugated goat anti-mouse and goat anti-rabbit IgG antibodies were purchased from Santa Cruz Biotechnology (CA, USA). Rhodamine-conjugated phalloidin was purchased from Thermo Fisher (CA, USA). ARHGEF siRNA (siRNA ID stB0002589-1) and ARHGEF6 siRNA (siRNA ID stB0003925-1) and negative control siRNA were purchased from RiboBio (Guangzhou, China). Y27632 was purchased from Selleck Chemicals (TX, USA). NSC23766 was purchased from Merck Serono (Darmstadt, Germany). Type I rat-tail collagen was purchased from Corning (NY, USA).

### Three-dimensional floating collagen matrix cultures

The three-dimensional collagen matrices were prepared as previously described [29]. First, collagen gels were prepared by mixing Type I rat-tail collagen (3.69 mg/mL), MEM (without serum) and an HSC suspension so that the final mixture resulted in a physiological ionic strength of MEM, 1 mg/mL collagen and 400,000 cells/mL. An 800-μL aliquot of the collagen gel solution was then added into each well of a 12-well culture plate. For floating collagen matrix cultures, the plate was incubated for 1 h at 37°C to allow gelation. Then, the collagen gel was detached from the well through gentle circumferential dislodgment using a 200-μL pipette tip and incubated at 37°C in a humidified atmosphere of 95% air and 5% CO_2_.

### RNA-Seq

Total RNA was isolated from the 2D PS- or 3D FCM-cultured HSCs using Trizol reagent (Invitrogen, USA). An Agilent 2100 Bioanalyzer (Agilent Technologies, USA) was used to evaluate the RNA quality, and the RNA integrity values for all samples were greater than 8.0. One microgram of RNA was used to generate a cDNA library based on the TruSeq RNA Sample Prep Kit v2 (Illumina, USA) according to the manufacturer’s instructions. Then, the cDNA libraries were sequenced on the Illumina HiSeq 2500 System (Illumina, USA) with 100 nucleotide paired-end reads per the standard manufacturer’s protocol. The RNA-Seq reads were aligned to the human reference genome (hg19) using Tophat (version 2.0.10 [30]) with default parameters. Read counts were scaled to reads per kilobase of exon model per million mapped reads (RPKM). Differentially expressed genes (DEGs) between 2D PS and 3D FCM cultured HSCs were determined using Student’s t-test. *P*-values < 0.05 and fold change > 2 were considered as the cutoff values for DEG screening. The extensively used DAVID software (Release 6.8β [31]) was selected to perform GO (cellular component) enrichment analysis. The statistical significance was determined using Fisher’s exact test followed by Benjamini correction; an adjusted *p* < 0.05 was considered significant.

### Immunofluorescence staining

For 2D immunofluorescence staining, cells grown on coverslips were washed 3 times in PBS, fixed for 15 min in 4% paraformaldehyde in PBS, and permeabilized for 10 min with 0.3% Triton X-100 in PBS. Nonspecific binding sites were blocked by 1 h incubation in 5% bovine serum albumin in PBS. After a 30 min wash with PBS, cells were incubated at 4 °C overnight with the primary antibody against Arp3 (1:200) to show the lamellipodia, as previously described [32]. After 3 × 5 min washes with PBS, cells were incubated with anti-rabbit Alex 488-conjugated secondary antibodies (1:200) for 1 h at room temperature. Following 3 × 5 min washes with PBS, the cells were incubated with rhodamine-conjugated phalloidin at 5 U/mL for 30 min. After additional PBS washes, the cells were mounted on a slide in mounting medium with DAPI (Molecular Probe, USA). Cells were examined and photographed with a confocal microscope (FV 300, Olympus).

For 3D immunofluorescence staining, cells were embedded in 3D FCM as described above. After 24 h, cells were fixed in 4% paraformaldehyde in PBS for 30 min and permeabilized for 30 min with 0.3% Triton X-100 in PBS. Cells were then incubated with rhodamine-conjugated phalloidin at 5 U/mL and DAPI for 2 h at room temperature and subsequently washed extensively with PBS. Cells completely embedded inside collagen gels were then imaged and photographed ≥ 150 μm above the surface with a confocal microscope (FV 300, Olympus).

### Transwell assay

For the migration assay, we used modified Boyden chambers with filter inserts (pore size, 8 μm). Approximately 5 × 10^4^ cells in 200 μL of MEM medium were placed in the upper chamber, and 1.5 mL of complete MEM medium was placed in the lower chamber. After 24 h in culture, cells were fixed in methanol for 15 min and then stained with 0.05% crystal violet in PBS for 15 min. Cells on the upper side of the filters were removed with cotton-tipped swabs, and the filters were washed with PBS. Cells on the underside of the filters were viewed and counted under a microscope.

### Stably transfected pEGF LX-2 cell selection and 3D collagen invasion assay

Cell transfection was performed by using Lipofectamine 3000 according to the manufacturer’s protocol. After 6 h of transfection, cells were treated with 400 μg/mL G418 sulfate (G418, Merck) for 14 d. pEGF LX-2 cells were selected by flow cytometry sorting. The 3D invasion assay was performed by using the OrisTM 3D Embedded Invasion assay kit (Platypus Technologies, LLC, WI, USA) following the manufacturer’s protocol.

### Real-time PCR

Total RNA was isolated using Trizol reagent (Invitrogen, USA). Two micrograms of RNA was reverse transcribed into single-strand cDNA in 20 μL of reaction buffer using a reverse transcriptase kit (Roche, USA). Primers were designed using the Primer 6 software (Table 2). GAPDH was chosen as an internal control. Thermal cycling conditions were as follows: 95 °C for 10 min, followed by 40 cycles of 95 °C for 30 sec and 60 °C for 1 min.

**Table 2.** Primers used for Real-time PCR.

Name		Sequences (5ʹ-3ʹ)	Product Size, bp
FAP	Forward	AAGTGTATGGTGGTCCCTGC	124
	Reverse	TGGAAAGCTGTTCCTCGACC	
LPXN	Forward	TGACTGAGATGCAGGCCAAG	82
	Reverse	GAGGCCTTGTGATCCTGCTT	
VCAM1	Forward	CTCCTGAGCTTCTCGTGCTC	98
	Reverse	TGACCCCTTCATGTTGGCTT	
SH3PXD2B	Forward	TCAGGTTGGTGGTTCGTCAG	117
	Reverse	CTTCTCCTCTTCTTCAGGCTGC	
MMP2	Forward	TGGCAAGTACGGCTTCTGT	179
	Reverse	TTCTTGTCGCGGTCGTAGTC	
MMP9	Forward	TGCGCTACCACCTCGAACTT	200
	Reverse	GATGCCATTGACGTCGTCCT	
ARHGEF4	Forward	CCTGCTTCCTGGAGCATCAA	172
	Reverse	AGGAAGCCATCCAGGGAGAT	
ARHGEF6	Forward	GACCATCAGCAGCACTAGGT	215
	Reverse	AAAATTTGCGCTGGTGCAGT	
GAPDH	Forward	GAGTCAACGGATTTGGTCGT	185
	Reverse	GACAAGCTTCCCGTTCTCAG	

### Western blotting

The cells were treated with lysis buffer (50 mM Tris-HCl pH 8, 150 mM NaCl, 5 mM ethylenediaminetetraacetic acid (EDTA), 1% NP40 and a protease inhibitor cocktail (Roche, USA) and phosphatase inhibitors (Merck, USA). To obtain HSCs cultured in 3D FCM, the collagen matrices were cut into small pieces and quickly digested with collagenase at a concentration of 10 mg/mL in a shaker within 5 min. The cells were washed with phosphate-buffered saline (PBS) several times and obtained after centrifugation. Equal quantities of protein were separated by 12% SDS-PAGE and transferred onto PVDF membranes (Amersham Biosciences) using a Bio-Rad wet transfer unit. After blocking with 5% (w/v) nonfat powdered milk in TBST solution (25 mM Tris, pH 7.5, 150 mM NaCl, 0.05% (v/v) Tween-20) for 1 h at room temperature, the membranes were incubated with primary antibody overnight at 4 °C, followed by horseradish peroxidase (HRP)-conjugated secondary antibody (1:5000) for 1 h at 37 °C. Target proteins were detected by use of Immobilon Western Chemiluminescent HRP Substrate (Millipore, US A).

### Statistics analysis

We used SPSS software version 18.0 (SPSS INC., Chicago, IL, USA) to conduct all statistical comparisons. The data are presented as the means ± SD. Nonparametric statistics were applied for comparison of results in all experiments unless stated otherwise. *P*-values less than 0.05 were considered statistically significant.
